# Protective Effects of the Traditional Herbal Formula Oryeongsan Water Extract on Ethanol-Induced Acute Gastric Mucosal Injury in Rats

**DOI:** 10.1155/2012/438191

**Published:** 2012-10-18

**Authors:** Woo-Young Jeon, Mee-Young Lee, In-Sik Shin, Hye-Sun Lim, Hyeun-Kyoo Shin

**Affiliations:** Basic Herbal Medicine Research Group, Korea Institute of Oriental Medicine, 483 Expo-ro, Yusung-gu, Daejeon 305-811, Republic of Korea

## Abstract

This study was performed to evaluate the protective effect and safety of Oryeongsan water extract (OSWE) on ethanol-induced acute gastric mucosal injury and an acute toxicity study in rats. Acute gastric lesions were induced via intragastric oral administration of absolute ethanol at a dose of 5 mL/kg. OSWE (100 and 200 mg/kg) was administered to rats 2 h prior to the oral administration of absolute ethanol. The stomach of animal models was opened and gastric mucosal lesions were examined. Gastric mucosal injuries were evaluated by measuring the levels of malondialdehyde (MDA), glutathione (GSH), and the activity of antioxidant enzymes. In the acute toxicity study, no adverse effects of OSWE were observed at doses up to 2000 mg/kg/day. Administration of OSWE reduced the damage by conditioning the gastric mucosa against ethanol-induced acute gastric injury, which included hemorrhage, hyperemia, and loss of epithelial cells. The level of MDA was reduced in OSWE-treated groups compared with the ethanol-induced group. Moreover, the level of GSH and the activity of antioxidant enzymes were significantly increased in the OSWE-treated groups. Our findings suggest that OSWE has a protective effect on the gastric mucosa against ethanol-induced acute gastric injury via the upregulation of antioxidant enzymes.

## 1. Introduction

It is well known that ethanol is metabolized mainly by alcohol dehydrogenases to form acetaldehyde, is then further metabolized to form acetate, and has toxic effects on the gastrointestinal tract [1]. Intake of ethanol induces the overproduction of reactive oxygen species (ROS) and the decrease in the activity of antioxidant enzymes, such as catalase (CAT), glutathione S-transferase (GST), glutathione peroxidase (GPx), superoxide dismutase (SOD), and glutathione reductase (GR), leading to gastric mucosal injuries, including ulceration, erosion, hemorrhage, congestion, and edema [2, 3].

As mentioned earlier, gastric damage caused by ethanol increases oxidative stress, leading to the excessive production of ROS, which is the main cause of oxidative stress. Overproduction of ROS plays a key role in the pathophysiological changes that occur in unsaturated fatty acids at the cell membrane, resulting in the increase of lipid peroxidation [4]. Hence, the measurement of lipid peroxidation via the determination of the concentration of MDA, the most widely used index of lipid peroxidation, possibly relates to the ability to scavenge oxygen free radicals [5]. To date, numerous antioxidants have been introduced to minimize the actions of ROS. For example, phenolic compounds can trap the free radicals directly or scavenge them through a series of coupled reactions with antioxidant enzymes. Previous studies reported that antioxidant enzymes reduce elevated levels of ROS via these enhancements [2, 5]. In addition, many studies have demonstrated that antioxidant enzymes exhibit a protective effect on ethanol-induced gastric mucosal injury using various experimental animals [2, 6]. In particular, Sprague-Dawley rats have been used in virtually all disciplines of biomedical research including toxicology and pharmacology. Ethanol-induced gastric lesions in rats are considered to be a reliable tool for studying the pathogenesis of acute gastric injury [7]. Ethanol-induced acute gastric lesions are characterized by pathological changes such as hemorrhage, edema, inflammatory infiltration, and loss of epithelial cells [8, 9]. Many researchers used SD rats as experimental animals to evaluate effect of herbal materials against acute gastric mucosal injury [5, 10]. Therefore, the present study focused on whether Oryeongsan has an antioxidative effect in an ethanol-induced gastric injury model.

Oryeongsan is a well-known mixed traditional herbal medicine used specifically for the treatment of renal diseases manifesting edema, dysuria, and oliguria [11]. It is composed of five herbs: Alismatis Rhizoma, Poria Sclerotium, Atractylodis Rhizoma Alba, Polyporus, and Cinnamomi Cortex (Table 1). According to some reports, Oryeongsan exhibits antihypertensive [12], antidiabetic [13], and antioxidative [14] effects and confers hepatic protection. However, despite these beneficial effects, research on Oryeongsan has not been actively pursued. Considering the properties of these herbs, we predicted that Oryeongsan water extract (OSWE) would decrease ethanol-induced acute gastric injury, possibly via antioxidative effects.

Acute toxicity is produced by the adverse effects of one or more doses of a substance, usually in less than 24 h. In addition, analysis of acute toxicity is often the basic step in the study of the safety of a substance [15]. Data from these tests can be used to screen for toxicity to determine if the OSWE is toxic. Therefore, we conducted an experiment to evaluate the protective effects and safety of OSWE on the ethanol-induced acute gastric mucosal injury and acute toxicity study in rats. The present study evaluates the scientific basis for the traditional use of OSWE.

## 2. Materials and Methods

### 2.1. Preparation of Oryeongsan

A voucher specimen of Oryeongsan (2008-KE17-1–KE17-5) is available at the Basic Herbal Medicine Research Group, Korea Institute of Oriental Medicine. Oryeongsan was prepared in our laboratory from a mixture of chopped crude herbs purchased from Omniherb (Korea) and HMAX (China, Vietnam). Professor Je-Hyun Lee of Dongguk University, Gyeongju, Republic of Korea, confirmed the identity of each crude herb. Oryeongsan was prepared as described in Table 1 and its extract was obtained by boiling the herbs in distilled water at 100°C for 2 h. The solution was evaporated and freeze-dried (yield, 22.7%). In HPLC analysis of Oryeongsan in a previous study, cinnamaldehyde and coumarin were determined as standard compounds [16]. As results of HPLC analysis, contents of standard compound in Oryeongsan were 3.683 mg/g and 1.103 mg/g, respectively.

### 2.2. Ethanol-Induced Gastric Injury

Specific-pathogen-free (SPF) male Sprague-Dawley (SD) rats weighing 200–250 g (aged 6 weeks) were purchased from Orient Co. (Seoul, Korea) and used after 1 week of quarantine and acclimatization. The animals were kept in a room at 23 ± 3°C with a relative humidity of 50% under a controlled 12 h/12 h light/dark cycle. The rats were given a standard rodent chow and sterilized tap water *ad libitum*. All experimental procedures were carried out in accordance with the NIH Guidelines for the Care and Use of Laboratory Animals and were approved by Korea Institute of Oriental Medicine Institutional Animal Care and Use Committee. The animals were cared for in accordance with the dictates of the National Animal Welfare Law of Korea.

Acute gastric lesions were induced via intragastric administration of absolute ethanol according to a method described previously [7]. The animals were divided into five groups (seven animals in each group): normal control (NC), ethanol (EtOH), omeprazole (Ome), and OSWE (OSWE-100, OSWE at 100 mg/kg of body weight; OSWE-200, OSWE at 200 mg/kg of body weight) groups, and fasted for 18 h before the experiment. Rats in the NC group were given PBS orally (5 mL/kg of body weight) as the vehicle, and the EtOH group received absolute ethanol (5 mL/kg of body weight) via oral gavage. Rats in the positive-control group were given oral omeprazole (50 mg/kg of body weight) 2 h prior to the administration of absolute ethanol. Omeprazole has been used widely for the treatment of gastritis because of its anti-inflammatory and antioxidant activities [17, 18]. Therefore, it was used as the positive-control drug in this study. The OSWE groups received OSWE (100 and 200 mg/kg of body weight, resp.) 2 h prior to absolute ethanol intake.

Animals in a group were sacrificed via cervical dislocation 1 h after receiving the absolute ethanol treatment. The stomach was removed from each animal and opened along its greater curvature. The tissue was gently rinsed in PBS. The stomach was stretched on a piece of cork with the mucosal surface facing upward and was then examined using a standard position for gross examination of gastric mucosal lesions. Photographs of hemorrhagic erosions in the stomach were acquired with a Photometrics Quantix digital camera. After the gastric lesions were photographed, the stomach tissue was cut in half and stored at −70°C for biochemical analysis.

### 2.3. Stomach Tissue Histopathology

The extent of mucosal injury was evaluated using light microscopy by an experienced histologist blinded to the treatment regimen. Quantitative analysis of gastric mucosal injury index was determined by the representative photographs using an image analyzer (Molecular Devices, Inc., CA, USA). Tissues were embedded in paraffin, sectioned at a thickness of 4 *μ*m, stained with H&E solution (hematoxylin, Sigma, MHS-16; and eosin, Sigma, HT1100-1-32), to measure the loss of epithelial cells and hemorrhage findings. Tissues were subsequently mounted and coverslipped using Dako mounting medium (Invitrogen Cooperation, CA, USA). The histopathological findings were assessed according to the criteria, as described previously [19, 20].

### 2.4. Biochemical Analysis

Stomach tissues were cut into small pieces and homogenized (1/10 w/v) with tissue lysis/extraction reagent containing a protease inhibitor (Sigma, MI, USA). The homogenates were centrifuged at 12,000 rpm for 10 min at 4°C, to discard any cell debris, and the supernatant was used to measure MDA, reduced GSH, CAT, GST, GPx, SOD, and GR. The concentration of total proteins was determined was using a protein assay reagent (Bio-Rad Laboratories, Inc.).

Lipid peroxidation was estimated via the determination of the level of MDA using a thiobarbituric acid reactive substance (TBARS) assay kit (BioAssay Systems, CA, USA). In brief, 100 *μ*L of homogenate was mixed with 100 *μ*L of 10% trichloroacetic acid and incubated for 15 min on ice. The mixture was centrifuged at 12,000 rpm for 5 min at 4°C. Subsequently, 200 *μ*L of supernatant was mixed with 200 *μ*L of thiobarbituric acid and incubated at 100°C for 60 min. The absorbance at 535 nm was measured after the mixture was cooled. The results are expressed as nmol of MDA/mg protein.

The contents of GSH were measured using a GSH assay kit (Cayman, MI, USA), and the results are expressed as *μ*mol/mg protein. The activity of antioxidant enzymes, including CAT, GST, GPx, SOD, and GR, was quantified using a commercial kit (Cayman, MI, USA) according to the manufacturer's protocols. The results are expressed as U/mg protein.

### 2.5. Acute Toxicity Study

 Male and female 5-week-old SD rats were purchased from an SPF facility at the Orient Bio Co. (Seoul, Republic of Korea) and used after 1 week of quarantine and acclimatization. All animals were housed in a room maintained at 23 ± 3°C with a relative humidity of 50%, artificial lighting from 08:00 to 20:00, and 10–20 air changes/h. The animals were fed a commercial pellet diet (PMI Nutrition International, Richmond, USA) and sterilized tap water *ad libitum* (after UV irradiation and filtration). The acute toxicity study was performed in compliance with the test guidelines of the Korea Food and Drug Administration (KFDA) under the Good Laboratory Practice Regulations for Nonclinical Laboratory Studies [21] and the study protocol was approved by the Institutional Animal Care and Use Committee of the Korea Institute of Toxicology (earned by AALAC International, 1998).

In the preliminary study, a single oral administration of OSWE did not induce any toxicity at a dose up to 2,000 mg/kg. Based on these results, the dose of 2,000 mg/kg was selected as the limit dose, as recommended by Organisation for Economic Co-operation and Development (OECD) test guidelines [22]. Ten rats of each sex were randomly assigned to two groups, with five rats in each group, and the animals received a single dose of 2,000 mg/kg via gavage. The vehicle-administered control rats received an equivalent volume of distilled water. After oral administration, all abnormal clinical signs were recorded before and after dosing at least twice a day, and body weight was measured on the day of dosing (day 1), immediately before treatment, as well as on days 2, 5, 8, and 15. At scheduled termination (day 15), all surviving animals were anesthetized by carbon dioxide and sacrificed by exsanguination from the aorta. Complete gross postmortem examinations were performed on all animals.

### 2.6. Statistical Analyses

Data are expressed as the mean ± standard deviation (SD). Significance was determined using analysis of variance (ANOVA). If the tests revealed the presence of a significant difference among the groups, the data were analyzed via a multiple comparison procedure using Dunnett's test [23]. Statistical analyses were performed using Path/Tox System (Ver. 4.2.2). The level of significance was set at *P* < 0.05 or 0.01.

## 3. Results

### 3.1. Acute Toxicity of OSWE

**Figure 1:**
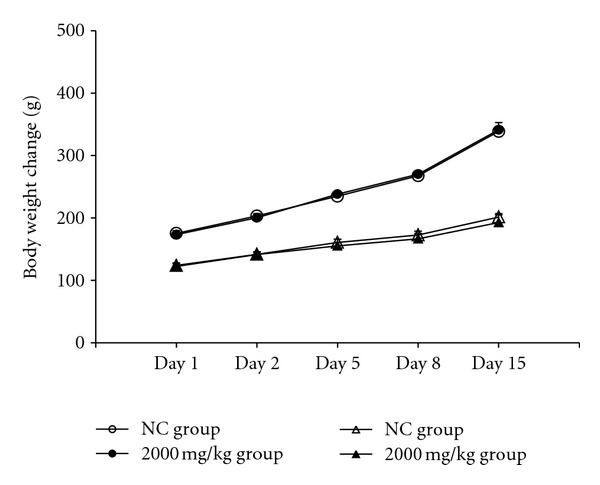
Body weight changes in animals treated with OSWE at dose levels of 0 mg/kg (◯) and 2,000 mg/kg (●) in males and 0 mg/kg (△) and 2,000 mg/kg (▲) in females. There were no significant differences in body weight between the OSWE-treated and control groups.

We evaluated the acute toxicity of OSWE, to investigate the safety of its oral administration. As shown in Figure 1, there were no significant differences in body weight changes between the OSWE-treated and NC groups for male and female rats. In addition, there were no observed clinical signs and gross findings in the OSWE-treated groups and NC groups, with the exception of loss of fur (*n* = 1) in the NC group (data not shown). 

### 3.2. Effect of OSWE on Ethanol-Induced Acute Gastric Injury

**Figure 2:**
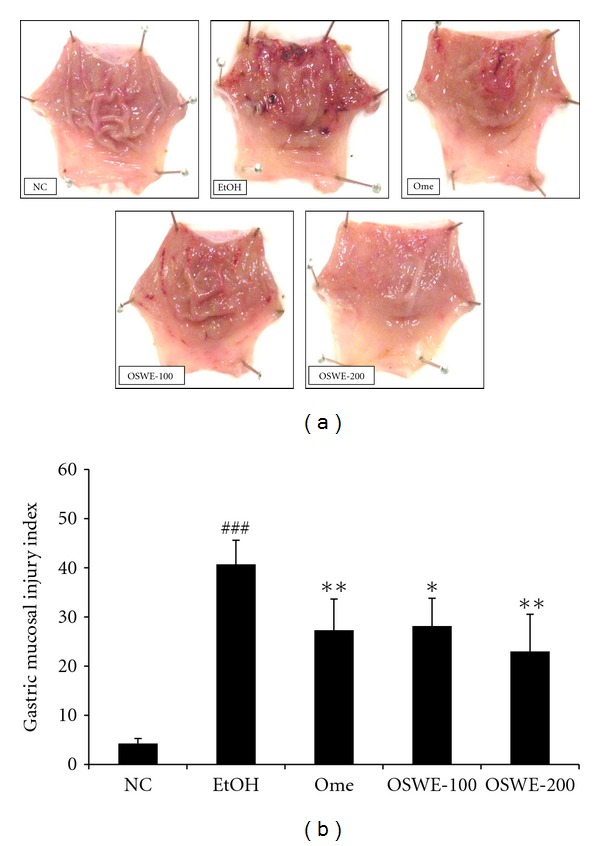
Representative photographs (a) and gastric mucosal injury index (b) of gastric mucosa with absolute-ethanol-induced gastric injuries. Gastric mucosal injury index (b) determined the findings of representative photographs using an image analyzer. NC: normal control group; EtOH: ethanol-induced group; Ome: EtOH + omeprazole (50 mg/kg)-treated group; OSWE-100: EtOH + OSWE (100 mg/kg)-treated group; OSWE-200: EtOH + OSWE (200 mg/kg)-treated group. Each bar represents the mean ± SD of seven rats. Significant differences at ^###^ *P* < 0.001 compared with the control group. Significant differences at *P < 0.05 and **P < 0.01 compared with the EtOH group.

**Figure 3:**
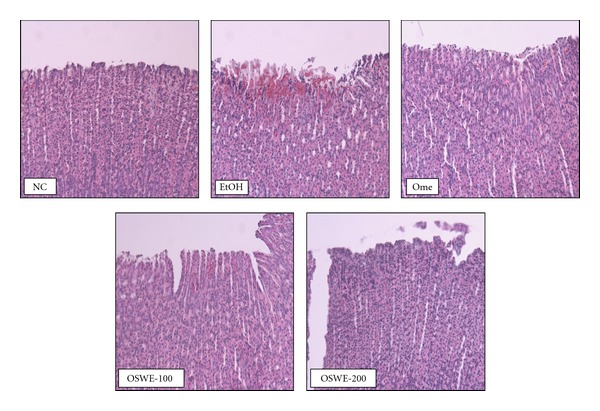
Histopathological examination of gastric mucosa with absolute-ethanol-induced gastric injury. NC: normal control group; EtOH: ethanol-induced group; Ome: EtOH + omeprazole (50 mg/kg)-treated group; OSWE-100: EtOH + OSWE (100 mg/kg)-treated group; OSWE-200: EtOH + OSWE (200 mg/kg)-treated group.

Although the EtOH group exhibited gastric mucosal injuries, including hemorrhage and hyperemia, the Ome group (which was used as positive control in this study) exhibited highly attenuated gastric mucosal injuries. Moreover, the OSWE-treated group showed a dose-dependent decrease in hemorrhage and hyperemia compared with the EtOH group (Figure 2(a)). In quantitative analysis, the OSWE-treated group indicated the dose-dependent and significant reduction in the gastric mucosal injury index compared with the EtOH (Figure 2(b)). The inhibition effect of OSWE was illustrated by histopathological examination. Loss of epithelial cells and hemorrhage findings were not detected in the stomach areas of the NC group. In contrast, the EtOH group displayed a marked loss of epithelial cells and hemorrhage findings. Treatment with OSWE at 100 or 200 mg/kg led to a reduction in the loss of epithelial cells and hemorrhage findings in the stomach area (Figure 3).

### 3.3. Effects of OSWE on MDA Concentration and GSH Levels

**Figure 4:**
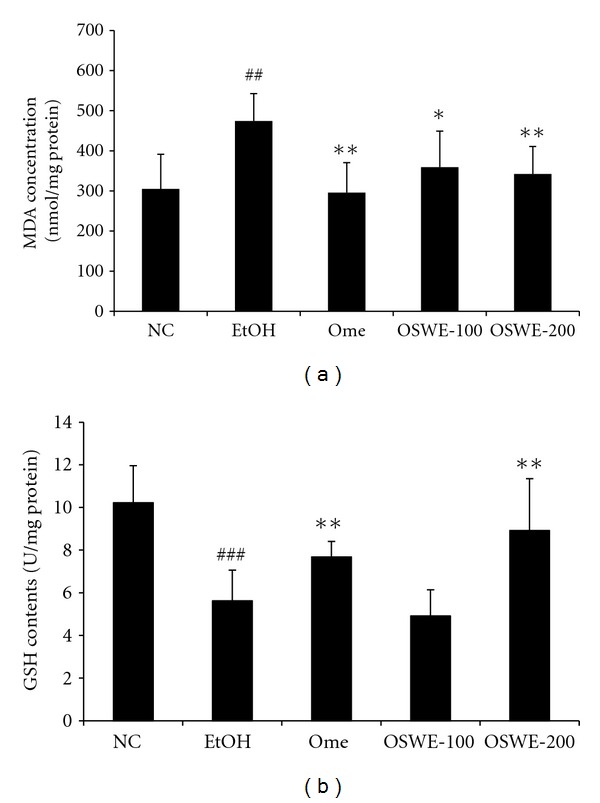
Effects of OSWE on gastric MDA concentration (a) and GSH levels (b) in rats with absolute-ethanol-induced gastric injury. NC: normal control group; EtOH: ethanol-induced group; Ome: EtOH + omeprazole (50 mg/kg)-treated group; OSWE-100: EtOH + OSWE (100 mg/kg)-treated group; OSWE-200: EtOH + OSWE (200 mg/kg)-treated group. Each bar represents the mean ± SD of seven rats. Significant differences at ^###^ *P* < 0.001 and ^##^ *P* < 0.01 compared with the control group. Significant differences at *P < 0.05 and **P < 0.01 compared with the EtOH group.

The level of MDA, an end product of lipid peroxidation, was significantly increased in the EtOH group compared with the NC group. However, the Ome group showed a significant decrease in MDA levels compared with the EtOH group. Moreover, the OSWE groups exhibited a significant dose-dependent decrease in the level of MDA compared with the EtOH group (Figure 4(a)). Conversely, the levels of GSH, a strong antioxidant, were significantly increased in the OSWE-200 group compared with the EtOH group (Figure 4(b)).

### 3.4. Effects of OSWE on the Activity of Antioxidant Enzymes

**Figure 5:**
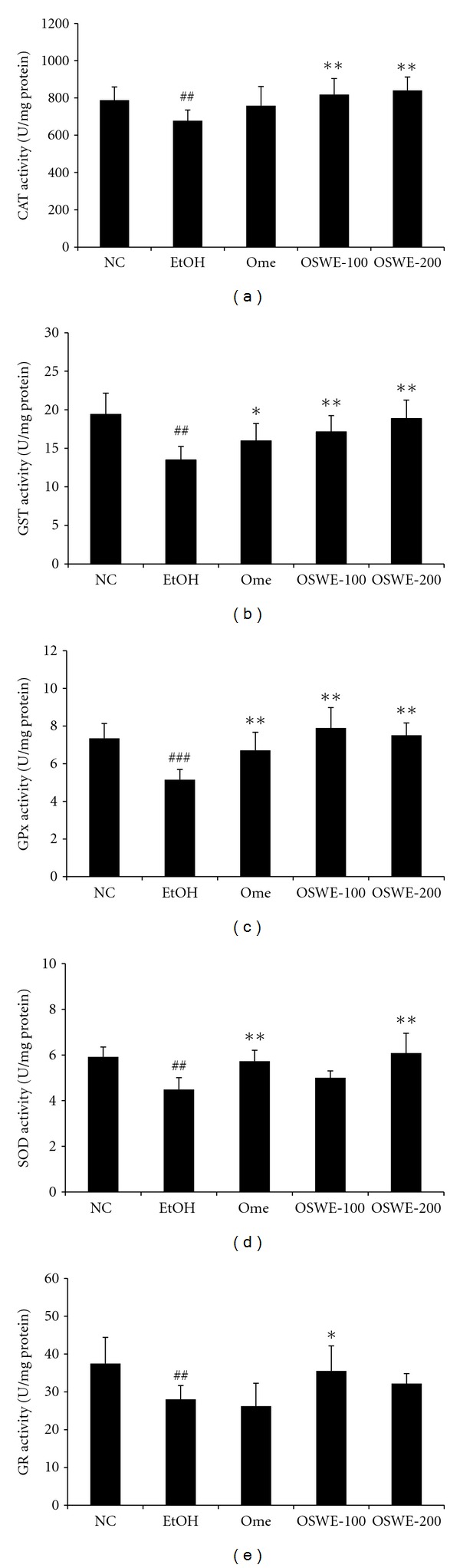
Effects of OSWE on the activity of gastric oxidative enzymes, including CAT (a), GST (b), GPx (c), GR (d), and SOD (e), in rats with absolute-ethanol-induced gastric injury. NC: normal control group; EtOH: ethanol-induced group; Ome: EtOH + omeprazole (50 mg/kg)-treated group; OSWE-100: EtOH + OSWE (100 mg/kg)-treated group; OSWE-200: EtOH + OSWE (200 mg/kg)-treated group. Significant difference at ^##^ *P* < 0.01 and ^###^ *P* < 0.001 compared with the control group. Significant difference at *P < 0.05 and **P < 0.01 compared with the EtOH group.

The activity of antioxidant enzymes, including CAT, GST, GPx, SOD, and GR, in the EtOH group was significantly decreased compared with the NC group. However, administration of OSWE significantly increased the ethanol-mediated decrease in the activity of these enzymes; these effects were similar to those observed for the NC group and were significantly different (CAT; *P < *0.01 in 100 and 200 mg/kg, GST; *P < *0.01 in 100 and 200 mg/kg, GPx; *P <* 0.01 in 100 and 200 mg/kg, SOD; *P <* 0.01 in 200 mg/kg, GR; *P <* 0.01 in 100 mg/kg) from those obtained for the group treated only with ethanol (Figures 5(a) and 5(e)). 

## 4. Discussion

The main purpose of the present study was to investigate whether OSWE has a protective effect on ethanol-induced acute gastric mucosal injury in the rat and whether it can be used safely (as assessed using an acute toxicity study). OSWE administration led to recovery from acute gastric injury (which included hemorrhage, hyperemia, and loss of epithelial cells) and to reduction of MDA concentration, increase of GSH levels, and enhancement of the activity of antioxidant enzymes.

The acute toxicity study is a basic step in the determination of the safety of materials. Our acute toxicity study, which was performed according to OECD guidelines, showed that OSWE is a safe material when administered to rats in a single dose via oral gavage, at a dose level of 2,000 mg/kg. In addition, clinical signs and gross findings of treatment-related adverse effects were not observed in any of the OSWE-treated Crl:CD (SD) rats (data not shown).

Ethanol is an ulcerogenic agent that is used often and produces severe gastric hemorrhagic lesions when given to rats via gavage [10]. Ethanol-induced acute gastric lesions are characterized by pathological changes such as hemorrhagic lesions, mucosal edema, inflammatory cell infiltration, and loss of epithelial cells [8, 24]. A previous study reported that the mechanism of ethanol-induced gastric lesions varies, including damaged mucosal blood flow and mucosal cell injury. In the present study, ethanol-induced gastric lesions were caused by severe gastric damage, including hemorrhage, hyperemia, and loss of epithelial cells. Several studies and our findings yielded similar results regarding ethanol-induced damage [2, 25]. However, administration of OSWE significantly attenuated the acute gastric injury induced by absolute ethanol. These results demonstrated that OSWE has a protective effect that occurs via the reduction of hemorrhage, hyperemia, and loss of epithelial cells that are associated with gastric mucosal injury. Similar to our results, earlier studies reported that the administration of an artichoke leaf extract confers gastroprotection by attenuating gastric lesions (including hemorrhage and hyperemia) [26].

In addition, ethanol-induced gastric mucosal injury is associated with overproduction of free radicals, which leads to increased lipid peroxidation [27, 28]. Lipid peroxidation is one of the major outcomes of free-radical-mediated injury, which immediately causes damage to the cell membrane and is related to DNA damage [29]. MDA, an end product of lipid peroxidation, is used as a marker of tissue damage [30]. Moreover, accumulation of MDA is considered a reliable biomarker of the degree of oxidative stress [31]. As mentioned above, in many studies, ethanol led to an increase in lipid peroxidation and a decrease in the activity of antioxidant enzymes, which are two of the factors that are important in the pathogenesis of ethanol-induced gastric injury. Our results showed that OSWE attenuated the increase in MDA concentration in the gastric mucosa injured by ethanol, thus indicating that OSWE can attenuate the process of lipid peroxidation implicated in the pathogenesis of ethanol-induced gastric damage.

Among the components of the OSWE herbal mix, we were able to estimate, based on previous studies, that the therapeutic effect of OSWE is exerted via the antioxidative effect of Alismatis Rhizoma [32], Poria Sclerotium [33], and Polyporus [34], the antiulcer effect of Atractylodis Rhizoma Alba [35], the anti-inflammatory effect of Cinnamomi Cortex [36]. In oriental herbal medicine, a mixture of several herbs is considered to enhance or prolong the pharmacological activities of any component herb and decrease its toxic effects [37]. Considering this theory, OSWE was considered to exhibit the synergic effects when each herb was formulated. Therefore, the present study focused on determining whether Oryeongsan has an antioxidative effect that is associated with a reduction of oxidative stress. However, we did not investigate the effects of each herb in OSWE against ethanol-induced gastric mucosal injury. Because of this reason, we did not evaluate the differences of beneficial effects between each herb and OSWE. In further study, we will conduct the studies on effects of active component herbs against ethanol-induced gastric mucosal injury.

Oxidative stress has been reported to be involved in many diseases. In addition, it is considered to cause gastric mucosal injuries. ROS production by oxidative stress is well controlled by antioxidant defenses under physiological conditions [38]. The antioxidant defense system includes GSH, CAT, GST, GPx, SOD, and GR, which are known as the main constituents of the intracellular protective mechanism that acts against oxidative injury [18].

GSH is a well-known antioxidant that is commonly present as the most abundant low-molecular-mass thiol in most organisms. It has multiple functions in the defense against oxidative stress [10]. Previous studies were performed to evaluate the gastroprotective effects of test drugs focusing on the alteration of GSH levels in gastric tissue [10, 25]. The present study showed that OSWE administration significantly increased the levels of GSH in gastric tissue damaged by ethanol. Thus, these results indicate that OSWE exhibits gastroprotective effects against ethanol-induced gastric damage via enhancement of GSH levels.

The antioxidant enzymes CAT and GPx play an important role in the detoxification of hydrogen peroxide (H_2_O_2_). GST consists of a group of isoenzymes that are capable of detoxifying various exogenous and endogenous substances by conjugation with glutathione. SOD, a family of enzymes, defends organisms against oxygen free radicals by catalyzing the elimination of the superoxide radical. This radical damages the cell membrane and other biological structures. GR is a glutathione regenerating enzyme that permits the conversion of oxidized glutathione (GSSG) to the reduced form (GSH) [5]. Reduced glutathione plays an important role in oxidoreduction processes and in the detoxification of H_2_O_2_ and organic peroxides [39]. Previous studies proved that the inhibition of oxidative stress occurs via the enhancement of the activities of enzymatic antioxidants [5, 40]. Our findings showed that OSWE treatment enhanced the activity of these antioxidant enzymes against ethanol-induced acute gastric injury. Therefore, these results suggest that OSWE exhibits antioxidative effects by increasing the activity of antioxidant enzymes.

## 5. Conclusion

The acute toxicity study performed here demonstrated that OSWE is a safe drug. In addition, this work revealed that OSWE reduced the concentration of MDA (which is related to lipid peroxidation) and gastric mucosal damage, including hemorrhage, hyperemia, and loss of epithelial cells. In contrast, OSWE administration increased the level of GSH and the activity of antioxidant enzymes. These results indicate that administration of OSWE has a protective effect against ethanol-induced gastric mucosal injury in rats. Therefore, OSWE may be considered a gastroprotective agent against oxidative injury caused by ethanol.

## Figures and Tables

**Table 1:** Crude components of Oryeong-san.

Scientific name	Amount (g)	Company of purchase	Source
Alismatis Rhizoma	9.375	Omniherb	Korea
Poria Sclerotium	5.625	Omniherb	Korea
Atractylodis Rhizoma Alba	5.625	Omniherb	China
Polyporus	5.625	HMAX	China
Cinnamomi Cortex	1.875	HMAX	Vietnam

Total amount	28.125		
